# Multifactorial determinants of health status: insights from the MEDIET4ALL large-scale survey on eco-sociodemographic, psychological, and lifestyle (diet, physical activity, and sleep) factors

**DOI:** 10.3389/fpubh.2026.1704240

**Published:** 2026-04-10

**Authors:** Achraf Ammar, Atef Salem, Mohamed Ali Boujelbane, Khaled Trabelsi, Bassem Bouaziz, Mohamed Kerkeni, Liwa Masmoudi, Juliane Heydenreich, Christiana Schallhorn, Gabriel Müller, Ayse Merve Uyar, Hadeel Ali Ghazzawi, Adam Tawfiq Amawi, Bekir Erhan Orhan, Giuseppe Grosso, Osama Abdelkarim, Tarak Driss, Kais El Abed, Piotr Zmijewski, Frédéric Debeaufort, Nasreddine Benbettaieb, Clément Poulain, Laura Reyes, Amparo Gamero, Marta Cuenca-Ortolá, Antonio Cilla, Nicola Francesca, Concetta Maria Messina, Enrico Viola, Björn Lorenzen, Stefania Filice, Sadjia Lahiani, Taha Khaldi, Nafaa Souissi, Omar Boukhris, Evelyn Frias-Toral, Haitham Jahrami, Waqar Husain, Walid Mahdi, Hamdi Chtourou, Wolfgang I. Schöllhorn

**Affiliations:** 1 Department of Training and Movement Science, Institute of Sport Science, Johannes Gutenberg-University Mainz, Mainz, Germany; 2 Research Laboratory, Molecular Bases of Human Pathology, LR19ES13, Faculty of Medicine of Sfax, University of Sfax, Sfax, Tunisia; 3 Interdisciplinary Laboratory in Neurosciences, Physiology, and Psychology: Physical Activity, Health, and Learning (LINP2), UFR STAPS, Paris Nanterre University, Nanterre, France; 4 Department of Nutrition and Food Technology, School of Agriculture, The University of Jordan, Amman, Jordan; 5 High Institute of Sport and Physical Education of Sfax, University of Sfax, Sfax, Tunisia; 6 Research Laboratory: Education, Motricity, Sport and Health, EM2S, LR19JS01, High Institute of Sport and Physical Education of Sfax, University of Sfax, Sfax, Tunisia; 7 Department of Movement Sciences and Sports Training, School of Sport Science, The University of Jordan, Amman, Jordan; 8 Multimedia InfoRmation Systems and Advanced Computing Laboratory (MIRACL), University of Sfax, Sfax, Tunisia; 9 Higher Institute of Computer Science and Multimedia of Sfax (ISIMS), University of Sfax, Sfax, Tunisia; 10 Department of Experimental Sports Nutrition, Faculty of Sports Sciences, Leipzig University, Leipzig, Germany; 11 Department of Sports Economics, Sociology, and History, Institute of Sport Science, Johannes Gutenberg-University Mainz, Mainz, Germany; 12 Faculty of Sports Sciences, Istanbul Aydın University, Istanbul, Türkiye; 13 Department of Biomedical and Biotechnological Sciences, University of Catania, Catania, Italy; 14 Department of Sports Management, School of Business, ESLSCA University, Giza, Egypt; 15 Institute of Sports and Sports Science, Karlsruhe Institute of Technology, Karlsruhe, Germany; 16 Faculty of Sport Sciences, Assiut University, Assiut, Egypt; 17 Department of Biomedical Sciences, Józef Piłsudski University of Physical Education in Warsaw, Warsaw, Poland; 18 Department Bioengineering, Institut Universitaire de Technologie IUT-Dijon-Auexrre-Nevers, University Burgundy Europe, Dijon, France; 19 Joint Research Unit UMR PAM-Food Processing and Microbiology, Université Bourgogne Europe, INRAE, Institut Agro Dijon, Dijon, France; 20 Vitagora Innovation Cluster, Dijon, France; 21 Department of Preventive Medicine and Public Health, Food Science, Toxicology and Forensic Medicine, Faculty of Pharmacy and Food Sciences, University of Valencia, Valencia, Spain; 22 Department of Agricultural Food and Forest Sciences SAAF, University of Palermo, Palermo, Italy; 23 Laboratory of Marine Biochemistry and Ecotoxicology, Department of Earth and Marine Sciences DiSTeM, University of Palermo, Trapani, Italy; 24 Microtarians SIS, Société d’Impact Sociétal, Luxembourg, Luxembourg; 25 VALCORE Laboratory, Department of Biology, Faculty of Science, University of M’hamed Bougara Boumerdes, Boumerdes, Algeria; 26 Biotechnology Research Center C.R.Bt Constantine ALGERIA, Constantine, Algeria; 27 Research Unit: Physical Activity, Sport, and Health, UR18JS01, National Observatory of Sport, Tunis, Tunisia; 28 SIESTA Research Group, School of Allied Health, Human Services and Sport, La Trobe University, Melbourne, VIC, Australia; 29 Sport, Performance, and Nutrition Research Group, School of Allied Health, Human Services and Sport, La Trobe University, Melbourne, VIC, Australia; 30 Escuela de Medicina, Universidad Espíritu Santo, Samborondón, Ecuador; 31 Division of Research, Texas State University, San Marcos, TX, United States; 32 Government Hospitals, Manama, Bahrain; 33 Department of Psychiatry, College of Medicine and Medical Sciences, Arabian Gulf University, Manama, Bahrain; 34 Department of Humanities, COMSATS University Islamabad, Islamabad, Pakistan

**Keywords:** dietary adherence, health predictors, mental well-being, non-communicable diseases, psychological distress, public health, sedentary behavior, socioeconomic disparities

## Abstract

**Background:**

Non-communicable diseases are a growing public health challenge, shaped not only by biological predispositions but also by geo-demographic, socioeconomic, psychological, and lifestyle factors. A comprehensive understanding of these determinants is essential for developing targeted public health strategies. This study aimed to examine the multifactorial determinants of individual health status by analyzing geo-demographic, socio-economic, behavioral, psychological, and lifestyle variables.

**Methods:**

Data were collected from 4,010 participants (age: 37.2 ± 15.4 years; 59.5% female) across 10 Mediterranean and neighboring countries using the multinational MEDIET4ALL e-survey. Health status was categorized as healthy, at-risk, or with diseases. Multinomial logistic regression, Quade’s Rank ANCOVA and series of multiple regression models were conducted.

**Results:**

Collectively, around 25% of respondents declared to be at-risk of or with known disease. BMI emerged as the strongest negative predictor of health status (*β* = −0.145), with both obesity and underweight significantly increasing the odds of being at risk (OR = 1.8 and 5.2, respectively) and having diseases (OR = 2.2 and 11.9, respectively). Other significant negative predictors included psychological distress (notably anxiety, *β* = −0.091), insomnia (*β* = −0.084), alcohol consumption (*β* = −0.053), and prolonged sitting time (*β* = −0.037). Conversely, life satisfaction was the strongest significant protective factor (*β* = 0.066), followed by higher education, better sleep quality, and adherence to the Mediterranean Diet and lifestyle (*β* = 0.034 to 0.050). Socio-economic disparities, including employment status (*β* = −0.045) and living environment (*β* = −0.031), also significantly influenced health outcomes with rural environment and employed individual showing lower odd ratios of being at-risk and/or having diseases (*p <* 0.001). Furthermore, individuals residing in Mediterranean regions, females, married or cohabiting individuals, and non-smokers exhibited significantly lower odds of being at-risk or having diseases (*p <* 0.05). While gender remained a significant predictor in the final refined comprehensive regression model (*β* = −0.049), marital status lost significance, suggesting that its protective effect may be mediated by psychological well-being and health-related behaviors.

**Conclusion:**

These findings highlight the complex interplay of lifestyle, mental health, and socio-environmental factors in determining health outcomes, while emphasizing the urgent need for multi-level public health interventions, including policies promoting physical activity, healthy eating, mental well-being, and equitable healthcare access. Future research should employ longitudinal designs to establish causal relationships and guides preventive strategies.

## 1 Introduction

The global health landscape faces significant challenges, particularly the growing burden of non-communicable diseases (NCDs) such as cardiovascular diseases, diabetes, obesity, and mental health disorders. According to the World Health Organization (1), these NCDs account for over 74% of global deaths, with this figure rising to 86% in Europe (2). The economic burden associated with NCDs are equally concerning, with Europe alone incurring healthcare costs exceeding €600 billion annually (Joint-Paper, 2019), further compounded by substantial productivity losses (3).

Understanding the root causes of these health issues is essential for effective intervention. NCDs are multifactorial, influenced not only by biological predispositions but also by socio-economic factors, environmental exposures, and lifestyle choices. Individuals from lower socioeconomic backgrounds are disproportionately affected by inadequate nutrition, limited access to healthcare, increased exposure to environmental pollutants, and chronic stress (4, 5). Additionally, demographic factors such as age, gender, and marital status interact with these socioeconomic determinants, further influencing NCD risk (5). For example, individuals with lower education levels and lower incomes exhibit higher prevalence rates of obesity, type 2 diabetes, cardiovascular diseases (CVD), and mental health disorders, largely due to disparities in preventive healthcare access, dietary habits, and physical activity (PA) levels (5). Economic hardship is also strongly linked to chronic stress, which can lead to physiological dysregulation, inflammation, dyslipidemia, and hypertension (6). Gender and marital status also influence health. Women in low-income households face higher obesity and metabolic risks due to food insecurity and restricted healthcare access (7), although recent evidence suggests that women may also show more health-conscious food choices as evidenced by better adherence to Mediterranean food consumption components (8). Widowed and divorced individuals, particularly older adults, experience greater NCD risk linked to reduced social support, physical activity and healthcare utilization (9–11).

Environmental determinants, such as urbanization, also contribute significantly to the rising burden of NCDs. Although urban areas may offer certain advantages, including more diverse food environments, structured PA opportunities, and better access to healthcare (12, 13), urbanization has also altered dietary habits in harmful ways, increasing exposure to ultra-processed foods and sugar-sweetened beverages that contribute to the global rise in obesity, type 2 diabetes, and cardiovascular diseases (14). Furthermore, limited green spaces, safety concerns, and sedentary occupations commonly reduce PA levels in urban settings, elevating risks of hypertension and obesity (13, 15).

Among the most concerning lifestyle trends is the increasing prevalence of sedentary behavior (16), compounded by poor dietary habits, particularly the shift away from traditional healthy diets toward ultra-processed food consumption (17, 18). Additionally, rising levels of social isolation and psychological stress further exacerbate health risks, as they have been associated with increased risks of cardiovascular disease, cognitive decline, and all-cause mortality (9).

Regular PA is recognized as one of the most crucial factors in preventing NCDs and their associated risk factors, such as overweight and obesity, while promoting overall well-being (19). Conversely, a sedentary lifestyle has been identified as a significant risk factor for various health problems (20). Recent data from the Global Burden of Disease study indicate that the contribution of low PA to deaths and disability-adjusted life years (DALYs) has increased by over 80% since 1990, accounting for approximately 1 million deaths and 16 million DALYs in 2019 (21). In contrast, reducing inactivity by just 10% could prevent over 530,000 deaths per year, and a 25% reduction could save over 1.3 million lives (16). Promoting PA is crucial not only in combating obesity and DALYs but also for mitigating mental health issues such as anxiety, depression, and cognitive decline (22, 23). Despite the well-documented benefits of PA, global adherence to recommended guidelines (i.e., at least 150 min of moderate PA or 75 min of vigorous-intensity PA per week for adults) remains low, with 27.5% of the global adult population failing to meet the recommended PA levels (24). This issue has been further exacerbated by the COVID-19 (25–27), and many individuals remained insufficiently active even after the pandemic (28, 29). Recent evidence from Mediterranean countries further shows that lifestyle and eating habits significantly influence physical activity levels among children and adolescents across five nations, underscoring the interconnected nature of diet, physical activity, and broader lifestyle patterns in shaping youth health (30).

Dietary habits are another lifestyle key determinant of health and well-being across the lifespan. The Mediterranean Diet (MedDiet), characterized by a high intake of fruits, vegetables, whole grains, and olive oil while limiting processed foods, is recognized as one of the most effective dietary models for promoting health and longevity while reducing NCD risk (31). Studies indicate that adherence to the MedDiet is associated with a 25% reduction in all-cause mortality (32) and significant protection against obesity, diabetes, cardiovascular diseases, and certain cancers (18). Moreover, the MedDiet aligns with environmental sustainability by promoting biodiversity, reducing greenhouse gas emissions, and supporting local food systems (33). Despite these benefits, adherence to the MedDiet has declined in recent years, even among adults and older adults living in Mediterranean regions (34, 35), largely due to the growing consumption of ultra-processed foods (14). The increasing reliance on ultra-processed foods has been linked to higher obesity rates, greater cardiovascular disease risk, and premature mortality (36). Recent evidence indicates that these dietary shifts are not limited to adults; findings from the DELICIOUS project show that ultra-processed food consumption is now widespread among children and adolescents in Mediterranean countries, with most consuming at least one ultra-processed food item daily across five nations (37, 38). These changes are driven by convenience, aggressive food marketing, variable levels of nutritional awareness, and shifts in food affordability (34, 35, 39).

Beyond sedentary behavior and unhealthy diets, psychosocial components such as psychological distress and social isolation are critical but often overlooked determinants of health (9, 40). Chronic stress, anxiety, and depression are major contributors to NCD risk, leading to increased inflammation, dyslipidemia, and hypertension (6, 41). Additionally, they often lead to unhealthy coping behaviors such as smoking, excessive alcohol consumption, and physical inactivity, further exacerbating NCD risk (42). Furthermore, loneliness and social isolation have been associated with adverse physical and mental health outcomes, including increased risks of cardiovascular disease, cognitive decline, and premature mortality (9, 40, 43). Despite the growing evidence supporting the detrimental effects of social isolation, recent reports from the EC indicated that in 2022, more than one third of the wide-EU survey respondents were lonely at least occasionally, with 13% reporting frequent loneliness (44). The COVID-19 pandemic further intensified social isolation and psychosocial distress (26, 45, 46), and many individuals have continued to experience reduced social engagement even after restrictions were lifted (47–49).

Taken together, evidence indicates that an individual’s health status is determined by a combination of environmental, socioeconomic, and lifestyle-related factors (4, 50). These findings underscore the need for integrated, multidomain public health policies that address these determinants while considering demographic factors such as age, gender, and marital status. Effective implementation of such strategies requires a comprehensive understanding of how health determinants interact and influence individual outcomes. However, most existing studies have examined single factors or limited interactions, leaving a gap for comprehensive multidimensional analyses (5, 51, 52). Recent studies also underscore the growing relevance of cross-country lifestyle surveillance, particularly in light of post-pandemic dietary shifts and widening sociodemographic disparities across the Mediterranean region (53, 54). Integrating such updated evidence ensures the contemporary relevance of the present investigation.

This study, conducted as part of the “MEDIET4ALL” PRIMA project (55) supported by the European Union, aims to bridge these gaps. By leveraging a large-scale survey approach across 10 Mediterranean and neighboring countries, this research aims to provide a comprehensive analysis of geo-demographic and socio-economic characteristics, psychological variables, and lifestyle behaviors associated with individual health status. Particularly, the study seeks to explore multifaceted differences between healthy, at-risk, and diseased populations, investigate the interplay between these determinants, and identify the most significant predictors of health status, while accounting for the confounding effects of age. The findings aim to inform targeted public health interventions and strategies that promote healthier and more sustainable lifestyles across diverse populations.

## 2 Methods

### 2.1 Survey development and participant recruitment

This cross-sectional study assessed adherence to MedDiet and mediterranean lifestyle (MedLife), along with potential determinant factors, using the MEDIET4ALL international electronic survey (8, 34, 35, 56). Prior to dissemination, the survey was subjected to a one-week pilot testing phase conducted by the project’s steering group. Subsequent revisions, informed by stakeholder feedback, were implemented to enhance clarity and validity. The finalized survey was subsequently administered over a four-month period commencing in the summer of 2024 across seven Mediterranean (Spain, Italy, France, Turkey, Tunisia, Algeria, and Morocco) and three adjacent countries (Germany, Luxembourg, and Jordan). Various organizations across Europe, North Africa, and Western Asia, supported both promotional efforts and data collection protocols. To ensure linguistic accessibility, the survey instrument was made available in seven languages: English, German, French, Italian, Spanish, Arabic, and Turkish. Reported internal consistency coefficients refer to previously validated versions of the respective instruments. For languages lacking pre-existing official translations, items underwent systematic translation and back-translation procedures in accordance with standardized methodological protocols. Test–retest reliability analyses conducted during the pilot phase demonstrated excellent stability for all translated items (r = 0.81–0.94), supporting the cross-linguistic applicability of the survey instruments.

The questionnaire comprised 75 items adapted from validated questionnaires, assessing MedLife adherence, identifying barriers to implementation, and evaluating contributing factors. These included geo-demographic and socioeconomic characteristics, health status, mental health, life satisfaction, and multidimensional lifestyle behaviors (e.g., PA, social engagement, sleep patterns, and technology utilization). The estimated completion time for the survey ranged between 15 and 20 min. The survey was administered via the SoSci Survey platform, a General Data Protection Regulation (GDPR)-compliant web application hosted through infrastructure provided by Johannes Gutenberg University. Distribution of the survey link was disseminated by the MEDIET4ALL consortium and affiliated partners (e.g., Bilendi Solutions) through various channels, including email invitations, official university and consortium web pages, the MEDIET4ALL website, and social media platforms such as ResearchGate™, LinkedIn™, Facebook™, WhatsApp™, and Twitter™. Additionally, members of the general public were encouraged to promote the survey within their personal networks.

Initial recruitment yielded a participant cohort exceeding 8,000 individuals from geographically diverse populations. Following systematic validity and completeness checks, 4,010 responses (~50% effective response/completion rate after quality checks) met inclusion criteria for final analysis. Completeness was operationally defined as submission of questionnaires with all mandatory items completed, as enforced by the SoSci Survey platform. Surveys with unanswered mandatory items could not be finalized and were therefore automatically excluded. Validity screening protocols included logic-based consistency checks to identify contradictory responses (e.g., concurrent denial of vigorous PA yet reporting daily strenuous exercise). Duplicate submissions were detected through analysis of IP address congruency, temporal proximity in submission timestamps, and homogeneity in demographic and substantive responses. Responses containing extreme or unrealistic values, such as reporting excessive sleep durations (e.g., 24 h) or implausible food intake patterns, were also removed to ensure data accuracy and reliability. When implausible or inconsistent values were identified, the entire participant record was excluded rather than applying data imputation procedures. This conservative approach was chosen to ensure data quality and analytical consistency. The sampling strategy prioritized broad population representativeness to maximize demographic diversity and analytical robustness.

### 2.2 Ethical considerations

The investigation adhered to ethical principles enshrined in the Declaration of Helsinki. Study protocols, including informed consent documentation, received formal approval from the Ethics Committee of the Faculty of Medicine at the University of Sfax (Approval Code: 058/24).

### 2.3 Data privacy and consent of participation

Participation in the study was strictly voluntary, with no justification required for declining involvement, withdrawing, or discontinuing participation at any stage. To uphold ethical standards, participants were explicitly advised that: (i) all data collected would be utilized exclusively for scientific research objectives, and (ii) responses would be anonymized and treated confidentially in alignment with the SoSci Survey privacy policy.^1^ The survey was administered via infrastructure hosted by Johannes Gutenberg University, ensuring compliance with the German Federal Data Protection Act (Bundesdatenschutzgesetz, BDSG) and the European Union’s General Data Protection Regulation (GDPR). No personally identifiable information (e.g., names, contact details) was solicited during the survey process. Participants retained the right to exit the survey at any time prior to final submission, with incomplete responses automatically discarded. Data were recorded solely upon active completion of the questionnaire, finalized by participant-initiated submission via the designated interface button. The study protocol guaranteed that withdrawal of consent or discontinuation of participation would incur no adverse consequences. Submission of the completed questionnaire constituted implicit informed consent for the anonymized use of participant data in accordance with the stated research objectives.

### 2.4 Survey questionnaires

The survey incorporated a variety of validated questionnaires as well as other specific questions to comprehensively assess adherence to the MedLifestyle, alongside associated factors.

#### 2.4.1 Depression anxiety stress scales-21 (DASS-21)

The DASS-21 serves as a psychometrically validated self-administered instrument designed to assess the severity of depressive, anxious, and stress-related symptomatology experienced within the preceding seven-day period (57). The tool partitions its 21 items equally across three distinct subscales, each corresponding to one of the aforementioned psychological constructs (Example item (Anxiety): *‘I felt I was close to panic.’*). Responses are recorded using a 4-point Likert scale (anchored from 0: did not apply to me to 3: applied to me very much, or most of the time). Raw subscale scores are aggregated and multiplied by a factor of two to align with the scoring protocol of the full-length DASS-42, thereby enabling severity classification into five ordinal categories: normal, mild, moderate, severe, or extremely severe (57). The DASS-21 demonstrates good-to-excellent internal consistency (subscale *α* typically ≥ 0.84) and a stable three-factor structure with acceptable short-term test–retest stability (58).

#### 2.4.2 Short life satisfaction questionnaire lockdown (SLSQ)

The SLSQ is a modified version of the Satisfaction with Life Scale (SWLS, showing strong internal consistency (α ≈ 0.87) and two-month test–retest reliability (r ≈ 0.82) (59)), adapted to include three items strongly associated with emotional well-being. Previously validated and used during the COVID-19 home confinement period (26, 45), the SLSQ enables individuals to make a conscious evaluative judgment of their life satisfaction using their own criteria (Example item: ‘I am satisfied with my life.’). Participants rated their agreement with each item on a 7-point Likert scale, ranging from 1 (“Strongly disagree”) to 7 (“Strongly agree”), with total scores ranging from 3 to 21. Higher scores indicate greater life satisfaction, categorized as follows: 3 (“Extremely dissatisfied”), 4–6 (“Dissatisfied”), 7–9 (“Slightly dissatisfied”), 10–12 (“Neutral”), 13–15 (“Slightly satisfied”), 16–18 (“Satisfied”), and 19–21 (“Extremely satisfied”).

#### 2.4.3 Sleep quantity, quality, latency and efficiency

A condensed adaptation of the Pittsburgh Sleep Quality Index (PSQI), that showed acceptable internal consistency (60), was employed to assess four sleep dimensions over the preceding month. While the full PSQI evaluates seven components, this version focuses on 4 selected items: Sleep Efficiency: Calculated as (total sleep time/time in bed) × 100; classified as >85% (optimal) or ≤85% (suboptimal); Sleep Latency: Categorized as <20 min (acceptable) or ≥20 min (prolonged); Subjective Sleep Quality (‘During the past month, how would you rate your sleep quality overall?’): Self-rated via a 4-point Likert scale (very good to very bad); Sleep Duration: Age-stratified as 7–9 h (<65 years) or 7–8 h (≥65 years) (61).

#### 2.4.4 Insomnia severity index (ISI)

The ISI is a validated psychometric instrument (62) operationalized as a self-report measure to quantify the severity and functional impact of insomnia symptomatology. Comprising seven items, it evaluates core clinical domains of insomnia, including: (i) difficulties initiating sleep, (ii) challenges maintaining sleep, (iii) premature awakening, (iv) subjective sleep satisfaction, (v) impairment in daytime functioning, (vi) perceptibility of sleep disturbances to others, and (vii) distress attributable to insomnia. Each item is scored on a 5-point Likert scale (0: no impairment; 4: severe impairment), yielding a cumulative score ranging from 0 to 28. Total scores are stratified into four clinical severity tiers (62): Absence of clinically significant insomnia (0–7); Subthreshold insomnia (8–14); Moderate insomnia (15–21); and Severe insomnia (22–28). The ISI shows strong internal consistency (*α* ≈ 0.90–0.91), acceptable test–retest reliability, sensitivity to treatment-related change, and in community samples a cutoff of 10 has reported sensitivity ~86% and specificity ~88% (62).

#### 2.4.5 Short social participation questionnaire -lockdowns (SSPQ)

The SSPQ is a brief, modified version of the Social Participation Questionnaire (SSPQ, acceptable internal reliability, with PSI: 0.71–0.74) designed to evaluate social participation behaviors over the past 12 months. It was previously validated and utilized during the COVID-19 home confinement period (26, 46). The questionnaire consists of 14 items (Example item: ‘Visited friends or relatives.’), with 10 rated on a 5-point scale from “never” to “all the time” and 4 requiring a binary “yes” or “no” response. The total SSPQ-L score is the sum of all responses, ranging from 14 to 70. A score of 14 indicates no social activity, while scores between 15 and 28 suggest rare social participation. Scores from 29 to 42 reflect occasional participation, 43 to 56 indicate frequent participation, and 57 to 70 represent consistent social engagement.

#### 2.4.6 International physical activity questionnaire short form (IPAQ-SF)

The IPAQ-SF is a self-reported questionnaire designed to assess PA levels over the past 7 days (high same-week test–retest reliability (Spearman *ρ* ≈ 0.80), covering different intensities such as vigorous, moderate, and walking activities (Example item: ‘During the last 7 days, on how many days did you do vigorous physical activities…?’ (63, 64). It measures total activity in MET-minutes per week (Metabolic Equivalent of Task) and classifies PA levels into three categories (low, moderate, and high) based on frequency, duration, intensity of activities, and/or calculated MET-minute/week (IPAQ, 2022). Widely used in both research and clinical settings, the IPAQ-SF facilitates the analysis of PA patterns across diverse populations and contributes to investigations of physical activity in relation to health outcomes.

#### 2.4.7 MedLife index

Existing MedDiet adherence indices were critically appraised in a systematic review by Zaragoza-Martí et al. (65), which identified 12 of 28 evaluated scores applicable to general populations. Among these, only the MEDLIFE Index, developed by Sotos-Prieto et al. (66), demonstrated rsobust internal consistency (Cronbach’s α = 0.75) and alignment with the MedDiet Pyramid framework (67, 68). Consequently, the MEDLIFE Index was selected for inclusion in the MEDIET4ALL e-survey (8, 34, 35, 56). This validated instrument assesses adherence to MedDiet and MedLife principles through 28 dichotomous items (scored 0: non-adherence; 1: adherence), aggregated into three domains: (i) Food Consumption Frequency (15 items): Evaluates intake patterns of core MedDiet components (e.g., fruits, vegetables, whole grains, healthy fats) and restricted foods (e.g., pastries, red meat) (Example Yes/No item: ‘≥2 servings/day of vegetables.’), (ii) MedDiet Habits (7 items): Examines behaviors such as salt/sugar reduction and avoidance of between-meal snacking, and (iii) Lifestyle Behaviors (6 items): Quantifies PA (≥150 min/week moderate intensity), sleep duration (6–8 h), social engagement, and conviviality.

Total scores (range: 0–28) were stratified into tertile-derived adherence categories: low (<12), moderate (12–16), and high (>16).

#### 2.4.8 Short technology-use questionnaire-lockdowns (STuQL)

The STuQL is a validated questionnaire designed to evaluate technology use in relation to social participation, dietary habits, and PA. It was previously implemented during the COVID-19 home confinement period (25, 26). The questionnaire consists of three items, each rated on a 5-point scale from “never” to “all the time.” (Example item: ‘How often do you use technology to support dietary choices?’) Total scores range from 3 (minimal use) to 15 (extensive use), with intermediate scores indicating different levels of technological engagement.

#### 2.4.9 The mediterranean barriers questionnaire (MBQ)

As described in Ammar et al. (34, 35) and Boujelbane et al. (8, 56), MBQ assesses perceived obstacles to adherence to the MedDiet. It comprises 13 binary items (Yes/No) covering a wide range of barriers, including health-related, cultural, economic, motivational, and accessibility factors. The total score ranges from 0 to 13, with higher scores indicating greater perceived barriers.

#### 2.4.10 Additional questions

The survey incorporated specific questions to capture geo-demographic, socioeconomic, and health-related characteristics. Participants provided detailed information on age, gender, marital status, education level, employment status, living environment (urban, suburban, or rural), country of residence, ethnicity, and smoking habits. Body mass index (BMI) was computed from self-reported height and weight, with classifications defined according to standard WHO thresholds: underweight (<18.5 kg/m^2^), normal weight (18.5–24.9 kg/m^2^), overweight (25.0–29.9 kg/m^2^), and obese (≥30.0 kg/m^2^). Health status was categorized into three groups: healthy (no known current disease), at risk of disease, or currently living with a diagnosed disease. The “at risk” category was defined based on (i) the presence of two or more cardiovascular risk factors, including hypertension, dyslipidemia, high cholesterol, overweight/obesity, current smoking, or physical inactivity, or (ii) a history of cardiovascular disease (stroke, transient ischemic attack, myocardial infarction, angina pectoris, or peripheral arterial disease), or (iii) the presence of diabetes mellitus, or (iv) a combination of these conditions (34). For analysis purposes, age was considered both as a continuous variable and a categorical variable, with participants classified into young adults (18–35 years), middle-aged adults (36–55 years), and older adults (>55 years) (69).

### 2.5 Statistical analysis

Descriptive statistics were used to summarize the distribution of health status categories across geo-demographic and socioeconomic factors. All statistical analyses were performed using SPSS (version 25; Chicago, IL, USA). To assess the association between geo-demographic and socioeconomic categorical variables (Table 1) and health status while adjusting for age as a confounder, we applied the Mantel–Haenszel chi-square test, followed by multinomial logistic regression (MLR). First, the Mantel–Haenszel chi-square test was used to examine whether health status (healthy, at risk, and with diseases) varied significantly across geo-demographic and socioeconomic categories while stratifying for three age groups (18–35, 36–55, and >55 years). This test allowed to control for age as a categorical variable and assess overall differences in health status distributions. Since the Mantel–Haenszel test does not account for age as a continuous variable, MLR has been conducted to obtain a more precise estimation of associations. In the present model: Health status was used as the dependent variable (reference category: healthy); geographical and socio-demographic variables were included as independent variables; and age was entered as a continuous covariate to adjust for potential confounding effects. For each independent variable, odds ratios (ORs) and *p*-values were calculated to determine the likelihood of being categorized as “at risk” or “with diseases” relative to being healthy, while accounting for age.

**Table 1:** Geo-demographic and socioeconomic information categorized by health status.

Variables	Proportions				MLR results			Mantel–Haenszel chi-square test
	Healthy (ref)	At risk	With diseases	Total	Heath status	*p*-value	OR	
Country of living								
Covariate: age categories					At risk	0.562	10.055	18–35: *χ*^2^(18) = 83.530. *p <* 0.001 36–55: *χ*^2^(18) = 72.830. *p <* 0.001 > 55: *χ*^2^(18) = 45.350. *p <* 0.001 Total: *χ*^2^(18) = 176.210. *p <* 0.001
					With diseases	0.000	0.451	
Algeria	80 (55%)	35 (24%)	31 (21%)	146	At risk	0.000	0.282	
					With diseases	0.000	0.154	
France	377 (71%)	108 (20%)	48 (9%)	533	At risk	0.041	0.551	
					With diseases	0.045	0.588	
Germany	410 (66%)	134 (22%)	72 (12%)	616	At risk	0.004	0.458	
					With diseases	0.000	0.386	
Italy	543 (77%)	116 (16%)	52 (7%)	711	At risk	0.036	0.544	
					With diseases	0.529	0.848	
Luxembourg	70 (59%)	34 (29%)	14 (12%)	118	At risk	0.190	0.596	
					With diseases	0.010	0.388	
Tunisia	132 (78%)	29 (17%)	9 (5%)	170	At risk	0.627	0.806	
					With diseases	0.580	0.799	
Spain	199 (72%)	65 (23%)	14 (5%)	278	At risk	0.724	1.142	
					With diseases	0.997	1.002	
Morocco	131 (82%)	23 (14%)	6 (4%)	160	At risk	0.953	0.971	
					With diseases	0.925	1.045	
Turkey	513 (86%)	66 (11%)	17 (3%)	596	At risk	0.951	0.979	
					With diseases	0.145	1.600	
Jordan	559 (82%)	98 (14%)	25 (4%)	682	At risk	.	.	
					With diseases	.	.	
Region								
Covariate: Age categories					At risk	0.931	1.008	18–35: *χ*2(2) = 11.063, *p =* 0.004 36–55: *χ*2(2) = 18.868, *p <* 0.001 > 55: *χ*2(2) = 2.568, *p =* 0.277 Total: *χ*2(2) = 3.826, *p =* 0.148
					With diseases	0.000	0.445	
Mediterranean	1976 (76%)	442 (17%)	177 (7%)	2,594	At risk	0.001	0.833	
					With diseases	0.000	0.752	
Non- Mediterranean	1,039 (73%)	266 (19%)	111 (8%)	1,416	At risk	.	.	
					With diseases	.	.	
Ethnicity								
Covariate: Age categories					At risk	0.585	10.052	18–35: *χ*^2^(14) = 52.80, *p <* 0.001 36–55: *χ*^2^(14) = 41.40. *p <* 0.001 > 55: *χ*^2^(14) = 24.28, *p =* 0.042 Total: *χ*^2^(14) = 122.043 *p <* 0.001
					With diseases	0.000	0.461	
Prefer not to say	128 (64%)	57 (28%)	16 (8%)	201	At risk	0.986	1.008	
					With diseases	0.526	1.330	
Black/African/Caribbean	109 (87%)	11 (9%)	5 (4%)	125	At risk	0.515	0.651	
					With diseases	0.226	2.025	
Latin American/Hispanic	44 (71%)	12 (19%)	6 (10%)	62	At risk	0.388	0.582	
					With diseases	0.777	0.852	
White/European	1,379 (71%)	381 (20%)	180 (9%)	1940	At risk	0.196	0.604	
					With diseases	0.660	1.175	
Asian	72 (65%)	20 (18%)	18 (16%)	110	At risk	0.024	0.325	
					With diseases	0.064	0.438	
Middle Eastern/Arab	743 (82%)	133 (15%)	30 (3%)	906	At risk	0.529	1.311	
					With diseases	0.036	2.330	
Turks	463 (84%)	63 (11%)	24 (4%)	550	At risk	0.558	0.769	
					With diseases	0.076	2.086	
Other	76 (66%)	31 (27%)	9 (8%)	116	At risk	.	.	
					With diseases	.	.	
Living environment								
Covariate: Age categories					At risk	0.960	0.996	18–35: *χ*^2^(4) = 5.291, *p =* 0.259 36–55: *χ*^2^(4) = 14.058, *p =* 0.007 > 55: *χ*^2^(4) = 3.938, *p =* 0.414 Total: *χ*^2^(4) = 44.098, *p <* 0.001
					With diseases	0.000	0.436	
Urban environment	2071 (78%)	422 (16%)	165 (6%)	2,658	At risk	0.098	1.333	
					With diseases	0.000	1.719	
Suburban environment	527 (73%)	148 (20%)	51 (7%)	726	At risk	0.058	1.513	
					With diseases	0.028	1.548	
Rural environment	416 (66%)	138 (22%)	72 (12%)	626	At risk	.	.	
					With diseases	.	.	
Gender								
Covariate: Age categories					At risk	0.651	0.962	18–35: *χ*^2^(2) = 3.13, *p =* 0.209 36–55: *χ*^2^(2) = 6.556, *p =* 0.038 > 55: *χ*^2^(2) = 1.972, *p =* 0.373 Total: *χ*^2^(2) = 3.871, *p =* 0.144
					With diseases	0.000	0.413	
Male	1,221 (75%)	301 (19%)	103 (6%)	1,625	At risk	0.046	1.336	
					With diseases	0.015	1.376	
Female	1793 (75%)	407 (17%)	185 (8%)	2,385	At risk	.	.	
					With diseases	.	.	
BMI								
Covariate: Age categories					At risk	0.000	1.044	18–35: *χ*^2^(6) = 73.14, *p <* 0.001 36–55: *χ*^2^(6) = 51.758, *p <* 0.001 > 55: *χ*^2^(6) = 40.602, *p <* 0.001 Total: *χ*^2^(6) = 235.894, *p <* 0.001
					With diseases	0.000	1.039	
Underweight:	162 (78%)	23 (11%)	24 (11%)	209	At risk	0.000	3.148	
					With diseases	0.305	1.276	
Normal weight	1790 (83%)	248 (12%)	108 (5%)	2,146	At risk	.	.	
					With diseases	.	.	
Overweight	1,051 (65%)	412 (26%)	151 (9%)	1,614	At risk	0.000	1.817	
					With diseases	0.000	2.224	
Obesity	11 (27%)	25 (61%)	5 (12%)	41	At risk	0.003	5.242	
					With diseases	0.000	11.866	
Education								
Covariate: Age categories					At risk	0.000	1.042	18–35: *χ*^2^(6) = 16.681, *p =* 0.011 36–55: *χ*^2^(6) = 26.575, *p <* 0.001 > 55: *χ*^2^(6) = 11.764, *p =* 0.067 Total: *χ*^2^(6) = 92.734, *p <* 0.001
					With diseases	0.000	1.043	
No schooling completed	157 (75%)	28 (13%)	23 (11%)	208	At risk	.	.	
					With diseases	.	.	
High school graduate, diploma or the equivalent/Professional degree	969 (67%)	328 (23%)	147 (10%)	1,444	At risk	0.539	0.859	
					With diseases	0.053	1.533	
Bachelor’s degree	1,178 (81%)	210 (14%)	67 (5%)	1,455	At risk	0.004	0.463	
					With diseases	0.428	1.197	
Master-doctorate degree	710 (79%)	142 (16%)	51 (6%)	903	At risk	0.004	0.454	
					With diseases	0.869	1.039	
Marital status								
Covariate: Age categories					At risk	0.000	1.044	18–35: *χ*^2^(4) = 97.015, *p <* 0.001 36–55: *χ*^2^(4) = 20.473, *p <* 0.001 > 55: *χ*^2^(4) = 19.479, *p =* 0.001 Total: *χ*^2^(4) = 174.607, *p <* 0.001
					With diseases	0.000	1.045	
Single	1,616 (82%)	258 (13%)	99 (5%)	1973	At risk	0.330	1.168	
					With diseases	0.160	1.162	
Married living as couple	1,255 (72%)	357 (20%)	134 (8%)	1746	At risk	.	.	
					With diseases	.	.	
Widowed, Divorced, Separated	143 (49%)	93 (32%)	55 (19%)	291	At risk	0.000	2.908	
					With diseases	0.000	1.837	
Employment								
Covariate: Age categories					At risk	0.000	1,043	18–35: *χ*^2^(8) = 54.038, *p <* 0.001 36–55: *χ*^2^(8) = 37.15, *p <* 0.001 > 55: *χ*^2^(8) = 31.868, *p =* 0.001 Total: *χ*^2^(8) = 300.999, *p <* 0.001
					With diseases	0.000	1,034	
Employed	1,556 (77%)	342 (17%)	135 (7%)	2033	At risk	.	.	
					With diseases	.	.	
Unemployed	317 (68%)	98 (21%)	52 (11%)	467	At risk	0.000	2.213	
					With diseases	0.000	1.604	
Student	898 (87%)	90 (9%)	39 (4%)	1,027	At risk	0.841	1.043	
					With diseases	0.141	0.810	
Retired	144 (42%)	143 (42%)	52 (15%)	339	At risk	0.036	1.585	
					With diseases	0.000	2.081	
Uncategorized	99 (69%)	35 (24%)	10 (7%)	144	At risk	0.802	1.091	
					With diseases	0.043	1.525	
Smoking								
Covariate: Age categories					At risk	0.000	2.371	18–35: *χ*^2^(2) = 9.180, *p =* 0.010 36–55: *χ*^2^(2) = 8.578, *p =* 0.014 > 55: *χ*^2^(2) = 0.532, *p =* 0.767 Total: *χ*^2^(2) = 10.16, *p =* 0.006
					With diseases	0.000	2.333	
Smokers	729 (72%)	210 (21%)	65 (7%)	1,004	At risk	0.017	1.518	
					With diseases	0.803	1.041	
Non-smokers	2,285 (76%)	498 (17%)	223 (7%)	3,006	At risk	.	.	
					With diseases	.	.	

**Figure 1:**
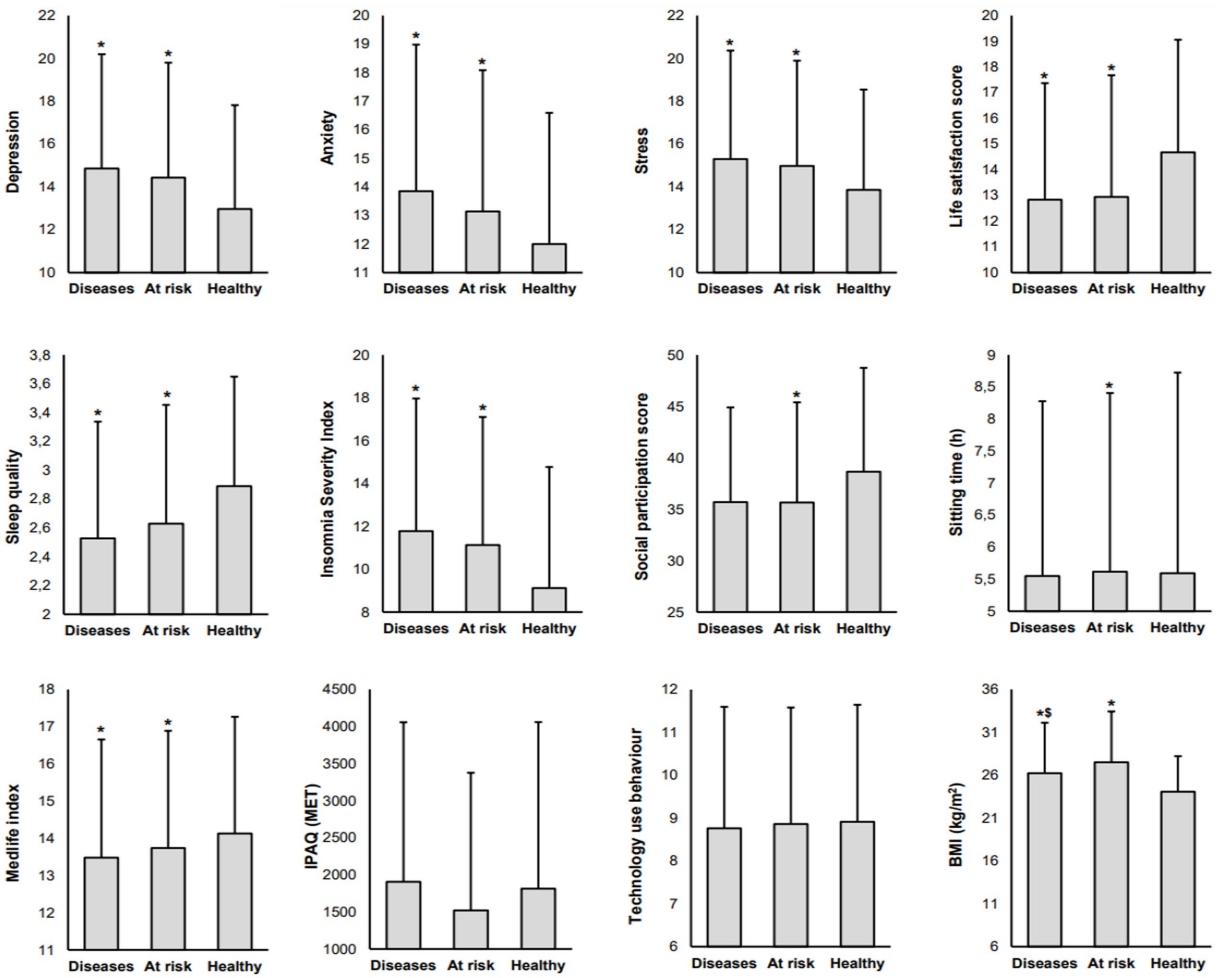
Mean and standard deviations of psychological well-being indicators and lifestyle behavior scores among participants stratified by health status (diseased, at risk, healthy). ^*^Significant difference compared to healthy participants; ^$^Significant difference compared to participants at risk.

The analysis was extended to examine the differences in consumer behavior and psychological state between different health status (Figure 1). Normality was assessed using the Shapiro–Wilk test, which indicated non-normal distributions. Given the test’s sensitivity in large samples, this assessment was complemented by visual inspection of histograms and Q–Q plots. As a result, Quade’s Rank ANCOVA, with age as a covariate, was applied to compare differences between health status groups. When significant effects were found, Dunn’s *Post-hoc* test with Bonferroni adjustment was performed to identify specific group differences. To identify the key determinants of health status (Tables 2, 3), a series of multiple regression models were conducted, each assessing the contribution of specific categories of predictors while adjusting for age, a common confounder variable in health research. The analysis followed a two-block approach, where Block 1 included age as a control variable in all models to adjust for its potential confounding effect, and Block 2 added different sets of potential contributing factors to evaluate their independent impact on health status. In the first model, BMI was examined as an anthropometric factor after controlling for age. The second model assessed the contribution of sociodemographic and environmental factors. The third model incorporated psychological factors, while the fourth model explored sleep-related factors. The fifth model considered physical and social activity factors with technology use behavior. The sixth model examined dietary factors as potential determinants of health status. Following these category-specific models, a comprehensive model (model 7) was developed, incorporating all significant variables identified from model 1 to model 6 to assess their combined impact on health status. A final refined comprehensive model (model 8) was then conducted, retaining only the significant predictors from model 7 to ensure parsimony while maintaining predictive power. Model performance was evaluated using multiple statistical criteria, including Adjusted 𝑅^2^ to assess the proportion of variance explained, *Δ*𝑅^2^ to measure the additional variance explained by each block beyond age, standardized beta coefficients (𝛽) to determine the relative strength of each predictor, and t-values and *p*-values to establish statistical significance. Semi-partial correlations (R) were examined to assess the unique contribution of each predictor to the dependent variable while controlling for the other variables in the model. The overall significance of each regression model was tested using ANOVA *F*-values and associated p-values to determine whether the predictors collectively explained a significant portion of the variance in health status. Additionally, ANOVA F-change and p-change values were analyzed to evaluate whether the inclusion of additional predictors in Block 2 led to a statistically significant improvement in model fit. Variance Inflation Factors (VIF) and tolerance values were examined to assess multicollinearity and ensure predictor independence.

**Table 2:** Potential predictive role of socio-demogarphic, psychological and behaviorals factors in health status.

Models/blocks		Factors	UC		SC	*t*	sig	R (semi-partial)	Collinearity statistics		SSE	Adj. R^2^	ANOVA		Change statistics		
			*b*	SE	*β*				Tolerance	VIF			F	*p*-value	R^2^ change	F change	*p*-change
Model 1	Block 1	(Constant)	3.089	0.024		129.416	<0.001				0.58	0.079	343.7	<0.001			
		Age	−0.011	0.001	−0.281	−18.540	<0.001	−0.281	1.000	1.000							
	Block 2	(Constant)	3.527	0.048		73.828	<0.001										
		Age	−0.009	0.001	−0.234	−14.983	<0.001	−0.224	0.918	1.090	0.57	0.103	232.0	<0.001	0.025	111.0	<0.001
		BMI	−0.020	0.002	−0.164	−10.534	<0.001	−0.158	0.918	1.090							
Model 2	Block 1	(Constant)	3.089	0.024		129.416	<0.001				0.58	0.079	343.7	<0.001			
		Age	−0.011	0.001	−0.281	−18.540	<0.001	−0.281	1.000	1.000							
	Block 2	(Constant)	3.121	0.061		50.756	<0.001										
		Age	−0.009	0.001	−0.234	−12.167	<0.001	−0.183	0.614	1.629							
		Gender	−0.045	0.019	−0.036	−2.398	0.017	−0.036	0.981	1.020							
		Marital status	−0.049	0.018	−0.051	−2.737	0.006	−0.041	0.660	1.515	0.57	0.93	59.63	<0.001	0.015	11.39	<0.001
		Employment	−0.026	0.008	−0.051	−3.289	0.001	−0.049	0.953	1.049							
		Ethnicity	0.007	0.006	0.020	1.252	0.211	0.019	0.905	1.105							
		Education	0.036	0.007	0.078	5.046	<0.001	0.076	0.941	1.062							
		Living environment	−0.033	0.012	−0.041	−2.651	0.008	−0.040	0.940	1.064							
Model 3	Block 1	(Constant)	3.089	0.024		129.416	<0.001				0.58	0.079	343.7	<0.001			
		Age	−0.011	0.001	−0.281	−18.540	<0.001	−0.281	1.000	1.000							
	Block 2	(Constant)	3.211	0.058		55.420	<0.001										
		Age	−0.012	0.001	−0.312	−20.651	<0.001	−0.305	0.958	1.044							
		Depression	−0.006	0.003	−0.052	−1.895	0.058	−0.028	0.295	3.394	0.56	0.124	114.53	<0.001	0.046	52.79	<0.001
		Anxiety	−0.016	0.003	−0.127	−5.135	<0.001	−0.076	0.358	2.791							
		Stress	0.000	0.003	0.004	0.144	0.885	0.002	0.312	3.206							
		SLSQ-L	0.040	0.006	0.100	6.214	<0.001	0.092	0.841	1.189							
Model 4	Block 1	(Constant)	3.089	0.024		129.416	<0.001				0.58	0.079	343.7	<0.001			
		Age	−0.011	0.001	−0.281	−18.540	<0.001	−0.281	1.000	1.000							
	Block 2	(Constant)	3.197	0.464		6.890	<0.001										
		Age	−0.012	0.001	−0.302	−19.724	<0.001	−0.292	0.937	1.067							
		Sleep latency	0.000	0.001	−0.018	−0.318	0.750	−0.005	0.065	15.302							
		Sleep duration	0.003	0.008	0.008	0.341	0.733	0.005	0.422	2.368	0.56	0.120	91.76	<0.001	0.042	38.18	<0.001
		sleep efficiency	0.000	0.005	−0.002	−0.026	0.979	0.000	0.060	16.564							
		Sleep quality	0.034	0.014	0.044	2.401	0.016	0.036	0.657	1.521							
		ISI	−0.017	0.002	−0.169	−8.969	<0.001	−0.133	0.615	1.625							
Model 5	Block 1	(Constant)	3.089	0.024		129.416	<0.001				0.58	0.079	343.7	<0.001			
		Age	−0.011	0.001	−0.281	−18.540	<0.001	−0.281	1.000	1.000							
	Block 2	(Constant)	3.038	0.061		49.637	<0.001										
		Age	−0.011	0.001	−0.270	−17.096	<0.001	−0.259	0.917	1.090							
		Sitting time	−0.010	0.003	−0.052	−3.434	<0.001	−0.052	0.996	1.004							
		IPAQ score	0.000	0.000	0.010	0.660	0.509	0.010	0.982	1.018	0.58	0.082	72.95	<0.001	0.005	4.916	<0.001
		SSPQ-L	0.002	0.001	0.038	2.403	0.016	0.036	0.901	1.110							
		Technology behavior	0.000	0.003	0.001	0.038	0.970	0.001	0.997	1.004							
Model 6	Block 1	(Constant)	3.089	0.024		129.416	<0.001				0.58	0.079	343.7	<0.001			
		Age	−0.011	0.001	−0.281	−18.540	<0.001	−0.281	1.000	1.000							
	Block 2	(Constant)	2.822	0.074		38.268	<0.001										
		Age	−0.011	0.001	−0.285	−18.810	<0.001	−0.283	0.985	1.015							
		MedLife index	0.018	0.003	0.096	6.207	<0.001	0.093	0.952	1.051	0.57	0.093	104.21	<0.001	0.015	22.52	<0.001
		MedDiet barriers	0.019	0.007	0.043	2.795	0.005	0.042	0.940	1.064							
		Alcohol	−0.078	0.015	−0.078	−5.145	<0.001	−0.077	0.992	1.008							

UC, unstandardized coefficients; SC, standardized coefficients; SEE, standard error of the estimate; R, semi-partial coefficient of correlation; R^2^, adjusted coefficient of determination; SLSQ-L, Score of Life Satisfaction Questionnaire; ISI, Insomnia Severity Index; IPAQ, The International Physical Activity Questionnaires; SSPQ-L, Short Social Participation Questionnaire.

**Table 3:** Comprehensive predictive models of health status.

Models/blocks		Factors	UC		SC	t	sig	R (semi-partial)	Collinearity statistics		SSE	Adj. R^2^	ANOVA		Change statistics		
			*b*	SE	*β*				Tolerance	VIF			F	*p-*value	R^2^ change	F change	*p*-change
Model 7	Block 1	(Constant)	3.089	0.024		129.416	<0.001				0.58	0.079	343.7	<0.001			
		Age	−0.011	0.001	−0.281	−18.540	<0.001	−0.281	1.000	1.000							
	Block 2	(Constant)	3.520	0.117		30.135	<0.001										
		Age	−0.009	0.001	−0.241	−12.560	<0.001	−0.181	0.562	1.779							
		BMI	−0.018	0.002	−0.143	−9.321	<0.001	−0.134	0.882	1.133							
		Gender	−0.057	0.018	−0.046	−3.080	0.002	−0.044	0.914	1.095							
		Marital status	−0.023	0.017	−0.024	−1.341	0.180	−0.019	0.651	1.536							
		Employment	−0.024	0.007	−0.048	−3.255	0.001	−0.047	0.947	1.056							
		Education	0.022	0.007	0.049	3.229	0.001	0.046	0.907	1.103							
		Living environment	−0.024	0.012	−0.030	−2.033	0.042	−0.029	0.948	1.054							
		Anxiety	−0.012	0.002	−0.092	−5.349	<0.001	−0.077	0.709	1.411	0.55	0.169	51.81	<0.001	0.092	29.876	<0.001
		SLSQ-L	0.009	0.002	0.065	4.055	<0.001	0.058	0.815	1.227							
		Sleep quality	0.035	0.014	0.045	2.539	0.011	0.037	0.654	1.529							
		ISI	−0.009	0.002	−0.085	−4.342	<0.001	−0.063	0.539	1.854							
		Sitting time	−0.007	0.003	−0.036	−2.501	0.012	−0.036	0.974	1.026							
		SSPQ-L	0.001	0.001	0.012	0.760	0.448	0.011	0.777	1.287							
		MedLife index	0.006	0.003	0.031	1.980	0.048	0.029	0.851	1.175							
		MedDiet barriers	−0.015	0.006	−0.035	−2.339	0.019	−0.034	0.929	1.076							
		Alcohol	−0.053	0.015	−0.053	−3.482	<0.001	−0.050	0.890	1.123							
Model 8	Block 1	(Constant)	3.089	0.024		129.416	<0.001				0.58	0.079	343.7	<0.001			
		Age	−0.011	0.001	−0.281	−18.540	<0.001	−0.281	1.000	1.000							
	Block 2	(Constant)	3.526	0.115		30.717	<0.001										
		Age	−0.010	0.001	−0.258	−16.164	<0.001	−0.233	0.817	1.224							
		BMI	−0.018	0.002	−0.145	−9.479	<0.001	−0.137	0.889	1.125							
		Gender	−0.060	0.018	−0.049	−3.268	0.001	−0.047	0.925	1.082							
		Employment	−0.023	0.007	−0.045	−3.072	0.002	−0.044	0.965	1.036							
		Education	0.023	0.007	0.050	3.306	<0.001	0.048	0.916	1.091							
		Living environment	−0.025	0.012	−0.031	−2.118	0.034	−0.031	0.952	1.051							
		Anxiety	−0.012	0.002	−0.091	−5.341	<0.001	−0.077	0.715	1.399							
		SLSQ-L	0.009	0.002	0.066	4.192	<0.001	0.060	0.849	1.177	0.55	0.169	59.04	<0.001	0.093	34.286	<0.001
		Sleep quality	0.036	0.014	0.047	2.639	0.008	0.038	0.659	1.518							
		ISI	−0.009	0.002	−0.084	−4.312	<0.001	−0.062	0.542	1.846							
		Sitting time	−0.007	0.003	−0.037	−2.517	0.012	−0.036	0.976	1.025							
		MedLife index	0.007	0.003	0.034	2.246	0.025	0.032	0.892	1.122							
		MedDiet barriers	−0.015	0.006	−0.035	−2.363	0.018	−0.034	0.930	1.075							
		Alcohol	−0.053	0.015	−0.053	−3.485	<0.001	−0.050	0.903	1.108							

UC, unstandardized coefficients; SC, standardized coefficients; SEE, standard error of the estimate; R, semi-partial coefficient of correlation; R^2^, adjusted coefficient of determination; SLSQ-L, Score of Life Satisfaction Questionnaire; ISI, Insomnia Severity Index; IPAQ, The International Physical Activity Questionnaires; SSPQ-L, Short Social Participation Questionnaire.

Significance was accepted for all analyses, *a priori*, at the level of *p <* 0.05.

## 3 Results

Data from 4,010 respondents (mean age: 37.2 ± 15.4 years; 59.5% female; mean weight: 71.3 ± 16.5 kg; mean height: 169.1 ± 9.9 cm) participating in the MEDIET4ALL survey were analyzed.

### 3.1 Association between geo-demographic and socioeconomic characteristics and health status of respondents

Table 1 presents the geo-demographic and socioeconomic characteristics of respondents categorized by health status. The Mantel–Haenszel chi-square test revealed significant associations between health status and most factors (*p <* 0.001), except for gender (*p* > 0.05). This association remained significant across all three age categories for country and region of residence, ethnicity, BMI category, and marital and employment status. However, for education level and smoking status, the association was only significant among young and middle-aged adults, while for living environment, it was observed only in the middle-aged group.

After adjusting for age as a continuous variable, the MLR results showed significant associations between health status and all factors. Significant differences in health risks emerged across countries. Compared to the reference category (Jordan), individuals from Algeria had significantly lower odds of being at risk (OR = 0.282, *p =* 0.000) and with diseases (OR = 0.154, *p =* 0.000). Similarly, individuals from France (OR = 0.551, *p =* 0.041), Germany (OR = 0.458, *p =* 0.004), and Italy (OR = 0.544, *p =* 0.036) had significantly reduced odds of being at risk. However, the association with disease prevalence was only significant for France (OR = 0.588, *p =* 0.045) and Germany (OR = 0.386, *p =* 0.000), suggesting a potential protective effect of healthcare systems in these countries. No significant associations were found for the remaining countries, except for disease prevalence in Luxembourg (OR = 0.388, *p =* 0.010). Regarding region of residence, individuals from mediterranean countries had lower odds of being at risk (OR = 0.833, *p =* 0.001) and with diseases (OR = 0.752, *p =* 0.000) compared to those from non-mediterranean countries, with significantly reduced odds for disease prevalence. The association between ethnicity and health status was not significant for most ethnic categories. However, individuals of Asian ethnicity had significantly lower odds of being at risk (OR = 0.325, *p =* 0.024) compared to the reference category (Other), while individuals of Middle Eastern/Arab ethnicity had significantly higher odds of being with diseases (OR = 2.330, *p =* 0.036). In terms of living environment, individuals residing in urban and suburban areas had higher odds of being at risk and with diseases compared to those in rural environments. However, this association was only statistically significant for disease prevalence, with p-values ranging from 0.028 to 0.000 and odds ratios (OR) between 1.548 and 1.719.

In contrast to the Mantel–Haenszel chi-square test, MLR analysis showed significant association with gender [*χ*^2^(2) 6.057, *p =* 0.048] with male having significantly higher odds of being at risk (OR = 1.336, *p =* 0.046) and with diseases (OR = 1.376, *p =* 0.015). Furthermore, BMI was strongly linked to deteriorating health, with overweight individuals displaying significantly higher odds of being at risk (OR = 1.817, *p =* 0.000) and with diseases (OR = 2.224, *p =* 0.000). Obesity was the most substantial predictor of poor health outcomes, with significantly elevated odds of being at risk (OR = 5.242, *p =* 0.003) and with diseases (OR = 11.866, *p =* 0.000). Additionally, underweight individuals showed notably high odds of being at risk (OR = 3.148, *p =* 0.000).

Education level appeared to have a protective role, particularly for those with a university degree. Individuals holding a bachelor’s degree as well as those with a master’s or doctorate degree had significantly lower odds of being at risk (OR range between 0.46, *p =* 0.004). Marital status also influenced health outcomes. Individuals who were widowed, divorced, or separated had higher risk of being at risk (OR = 2.908, *p =* 0.000) and developing diseases (OR = 1.837, *p =* 0.000). Employment status significantly influenced health outcomes, with unemployed individuals showing higher odds of being at risk (OR = 2.213, *p =* 0.000) and with diseases (OR = 1.604, *p =* 0.000). Similarly, retired individuals also faced an increased likelihood of being at risk (OR = 1.585, *p =* 0.036) and with diseases (OR = 2.081, *p =* 0.000). Smoking status was also associated with health risks, with cigarette smokers displaying a significantly higher likelihood of being at risk (OR = 1.518, *p =* 0.017) compared to non-smokers.

### 3.2 Comprehensive effect of MedLife adherence on various consumer behaviors

Figure 1 presents the differences in psychological status and lifestyle behaviors between the three different responders’ health status.

The analysis revealed a significant main association between Health and depression [*F*(2, 4,006) = 77.42, *p <* 0.001, *η*^2^ = 0.037], anxiety [*F*(2, 4,006) = 81.03, *p <* 0.001, *η*^2^ = 0.039], and stress [*F*(2, 4,006) = 62.47, *p <* 0.001, *η*^2^ = 0.03]. Additionally, Age was a significant covariate for depression, anxiety, and stress [*F*(1, 4,006) = 203.09, *p <* 0.001, *η*^2^ = 0.048; *F*(1, 4,006) = 270.81, *p <* 0.001, *η*^2^ = 0.063; *F*(1, 4,006) = 214.29, *p <* 0.001, *η*^2^ = 0.051; respectively]. *Post-hoc* comparisons showed that healthy participants had significantly lower depression, anxiety, and stress scores compared to participants with diseases (*p <* 0.001) and at risk (*p <* 0.001). Similarly, life satisfaction differ significantly between different health status [*F*(2, 4,006) = 58.88, *p <* 0.001, *η*^2^ = 0.029] and age was a significant covariance [*F*(1, 4,006) = 6.09, *p =* 0.014, *η*^2^ = 0.002], with healthy participants reporting significantly higher life satisfaction compared to those with diseases (*p <* 0.001) and those at risk (*p <* 0.001).

A similar trend was observed for sleep patterns, with both sleep quality [*F*(2, 4,006) = 46.5, *p <* 0.001, *η*^2^ = 0.023] and insomnia severity [*F*(2, 4,006) = 96.08, *p <* 0.001, *η*^2^ = 0.046] showing significant differences between the different health status. Age was a significant covariance for insomnia severity [*F*(1, 4,006) = 167.86, *p <* 0.001, *η*^2^ = 0.04], but not for sleep quality [*F*(1, 4,006) = 0.23, *p =* 0.633, *η*^2^ = 0]. Healthy participants reported significantly better sleep quality and lower insomnia severity compared to those with diseases (*p <* 0.001) and those at risk (*p <* 0.001).

Similarly, lifestyle related scores differ significantly between health status, including social participation [*F*(2, 4,006) = 4.11, *p =* 0.071, *η*^2^ = 0.002], sitting time [*F*(2, 4,006) = 8.7, *p <* 0.001, *η*^2^ = 0.004], and MedDiet adherence [*F*(2, 4,006) = 16.05, *p <* 0.001, *η*^2^ = 0.008]. Age was a significant covariance for social participation [*F*(1, 4,006) = 304.41, *p <* 0.001, *η*^2^ = 0.071] and MedDiet adherence [*F*(1, 4,006) = 33.73, *p <* 0.001, *η*^2^ = 0.01], but not for sitting time [*F*(1, 4,006) = 1.8, *p =* 0.18, *η*^2^ = 0]. Healthy participants exhibited higher adherence to the MedDiet compared to those at risk (*p <* 0.001) and those with diseases (*p <* 0.001). Additionally, healthy participants have significantly greater social participation and lower sitting time compared to participants at risk (*p <* 0.001). However, PA levels and technology use behaviors did not differ significantly by health status [*F*(2, 4,006) = 2.79, *p =* 0.061, *η*^2^ = 0.001 and *F*(2, 4,006) = 0.08, *p =* 0.919, *η*^2^ = 0, respectively]. Age was a significant covariance for technology use behaviors [*F*(1, 4,006) = 7.01, *p =* 0.008, *η*^2^ = 0.002], but not for physical activity levels [*F*(1, 4,006) = 0.69, *p =* 0.404, *η*^2^ = 0].

BMI differs by health status [*F*(2, 4,006) = 63.13, *p <* 0.001, *η*^2^ = 0.031], and age was significant covariate for BMI [*F*(1, 4,006) = 276.5, *p <* 0.001, *η*^2^ = 0.065]. Healthy participants reported a significantly lower BMI compared to participants with diseases (*p <* 0.001) and at risk (*p <* 0.001), and participants at risk were higher compared to participants with diseases (*p <* 0.001).

### 3.3 Potential predictors of adherence to the mediterranean lifestyle

Table 2 presents the results of six multiple regression models, each assessing the role of specific categories of predictors in explaining health status while adjusting for age. The explained variance across models, as indicated by Adjusted R^2^ values and change in R^2^ (ΔR^2^), varied, with psychological factors (Model 3) demonstrating the highest explanatory power (R^2^_adj = 0.124, ΔR^2^ = 0.046), followed by sleep-related factors (Model 4, R^2^_adj = 0.120, ΔR^2^ = 0.042), and BMI (Model 1, R^2^_adj = 0.103, ΔR^2^ = 0.025). Lower explanatory power was recorded for sociodemographic and environmental factors (Model 2, R^2^_adj = 0.093, ΔR^2^ = 0.015) and dietary patterns (Model 6, R^2^_adj = 0.093, ΔR^2^ = 0.015), with the least variance explained by physical and social activity factors (Model 5, R^2^_adj = 0.082, ΔR^2^ = 0.005). Despite differences in explanatory power, all models were statistically significant as indicated by their respective ANOVA *F*-values (*p <* 0.001) and F-change values (*p <* 0.001), confirming that the inclusion of predictors in Block 2 significantly improved the models beyond the age-adjusted baseline, which alone accounted for 7.9% of the variance in health status.

Model 3, which included psychological factors, accounted for 12.4% of the variance in health status, showing the greatest predictive power among all models. The model improvement was statistically significant (ΔR^2^ = 0.046, F_change = 52.79, *p <* 0.001). Among the psychological predictors, anxiety (*β* = −0.127, R = −0.076) had a significant negative association with health, while life satisfaction (SLSQ-L, *β* = 0.100,) was a significant positive predictor. The semi-partial correlations (R) further indicate that these two psychological variables (R = 0.092 and −0.076, respectively for life satisfaction and anxiety) had the strongest independent contribution in this model.

Model 4, focusing on sleep-related factors, demonstrated the second-highest explanatory power, accounting for 12.0% of the variance in health status (ΔR^2^ = 0.042, F_change = 38.18, *p <* 0.001). Insomnia severity (ISI, *β* = −0.169, R = −0.133) had the strongest negative association, while sleep quality (*β* = 0.044, R = 0.036) was positively associated with better health outcomes. Sleep duration, sleep efficiency, and sleep latency were not significant predictors.

Model 1, examining BMI, accounted for 10.3% of the variance (ΔR^2^ = 0.025, F_change = 111.0, *p <* 0.001), confirming that BMI is an important determinant of health. BMI (*β* = −0.164, R = −0.158) was a strong negative predictor, indicating that individuals with higher BMI tend to have poorer health status.

Model 2, which included sociodemographic and environmental factors, explained 9.3% of the variance (ΔR^2^ = 0.015, F_change = 11.39, *p <* 0.001). Gender, marital status, employment and living environment (*β* range between −0.036 and −0.051; R range between −0.36 and −0.49) showed significant negative associations, while education (*β* = 0.078, R = 0.076) had a significant positive contribution to health. Model 6, focusing on dietary patterns, also accounted for 9.3% of the variance (ΔR^2^ = 0.015, F_change = 22.52, *p <* 0.001). MedLife index (*β* = 0.096, R = 0.093) was a significant positive predictor, while dietary barriers (*β* = −0.043, R = −0.042) and alcohol consumption (*β* = −0.078, R = −0.077) were negatively associated with health. Model 5, examining physical and social activity factors, had the lowest explanatory power, accounting for 8.2% of the variance (ΔR^2^ = 0.005, F_change = 4.92, *p <* 0.001). Sitting time (*β* = −0.052, R = −0.052) had a significant negative association with health, while social participation (SSPQ-L, *β* = 0.038, R = 0.036) was a significant positive predictor.

Building on the analysis from Tables 2, 3 presents two comprehensive predictive models (model 7 and 8) that incorporate socio-demographic, environmental, psychological, sleep, PA, social engagement, and dietary variables. Both models explained 16.9% of the variance in health status (R^2^_adj = 0.169), indicating a significant improvement over previous models (*p <* 0.001, F_change = 29.88 and 34.29, respectively) with model 8 showing slightly parsimony improvement (ΔR^2^ = 0.092 and 0.093, respectively). Model 7, which incorporated all significant predictors from Models 1 to 6, indicates that the majority of potential predictors revealed in the category-specific models remain significant except Marital status and social participation (*p* > 0.05). The final refined model, Model 8, including only the significant variables from Model 7, revealed that BMI (*β* = −0.145, R = −0.137) remain the strongest negative predictors, followed by anxiety (*β* = −0.091, R = −0.077), insomnia severity (*β* = −0.084, R = −0.062), alcohol consumption (*β* = −0.053, R = −0.050), gender (*β* = −0.049, R = −0.047), employment (*β* = −0.045, R = −0.044) and lastly sitting time, MedDiet barriers and living environment (*β* = −0.031 to −0.037 and R = −0.031 to −0.036). Among the significant positive association, life satisfaction (*β* = 0.066, R = 0.060) emerged as the strongest predictors, followed by education (*β* = 0.050, R = 0.048), sleep quality (*β* = 0.047, R = 0.038), and adherence to MedDiet and lifestyle (*β* = 0.034, R = 0.032).

The findings from the multicollinearity test indicate that, with the exception of sleep latency and sleep efficiency (which were not significant predictors in Model 4), all other tested predictors across all models showed no relevant multicollinearity issues, with VIF values ranging from 1.0 to 3.39 and tolerance values between 0.295 and 0.997.

## 4 Discussion

This study aimed to comprehensively examine the multifactorial determinants of individual health status by integrating geo-demographic, socioeconomic, psychological, and lifestyle factors, while also accounting for the confounding effects of age. Using data from the MEDIET4ALL large-scale survey, we explored the interplay between these potential determinants and their influence on the health status of individuals categorized as healthy, at risk, or with diseases. The findings highlight the complex and interconnected nature of health determinants, revealing the significant influence of various geo-demographic and socioeconomic factors, as well as notable differences across health status categories in multiple behavioral and psychological scores. Notably, the refined comprehensive regression model highlighted the substantial influence of BMI, emerging as the strongest negative predictor, along with psychological distress, particularly anxiety, and poor sleep (insomnia severity). Additionally, unhealthy behaviors such as alcohol consumption and prolonged sitting time were also significantly associated with poorer health outcomes. Furthermore, gender, employment status, and living environment contributed to health disparities. Conversely, life satisfaction was the strongest protective factor, followed by higher education, better sleep quality, and adherence to the MedDiet and MedLife. This comprehensive model demonstrated greater explanatory power compared to individual category-specific models, suggesting that health outcomes are shaped by an intricate interplay of biological, psychological, behavioral, and socio-environmental factors, rather than any single determinant.

### 4.1 Geo-demographic and socioeconomic factors

The present MLR analysis revealed significant associations between all examined geo-demographic and socioeconomic factors and health status. Specifically, lower education levels, unemployment or retirement, widowed, divorced, or separated status, urban or suburban living, and male gender, were all associated with significantly higher odds of being at risk and/or having diseases. Conversely, higher education, employment, and residing in Mediterranean regions appeared to offer protective effects. Moreover, geographical disparities were evident, with individuals from certain European countries (e.g., Germany, France, and Italy) exhibiting lower odds of being at risk. Notably, across various regression models, gender, employment, education, and living environment factors showed significant contributions to MedLife adherence. However, marital status showed significant impact only in the individual category-specific models.

Consistent with previous evidence, our findings support the well-documented association between higher educational attainment and improved health outcomes, likely mediated by enhanced health literacy, increased access to preventive care, and healthier behaviors (5). Individuals with higher education levels are more likely to engage in regular PA, adhere to healthier diets, and access preventive healthcare services, thereby reducing their risk of developing chronic diseases such as CVDs, type 2 diabetes, and obesity (70). Education also mitigates the impact of economic instability, as higher-educated individuals typically have greater employment opportunities and financial security, which in turn facilitates access to healthcare and healthier lifestyle choices while reduce chronic stress (4, 5). Conversely, unemployment and retirement were significantly associated with higher health risks, supporting prior findings that economic instability is a strong predictor of chronic diseases (4, 5). Economic hardship is often linked to chronic stress, reduced healthcare access, poor diet quality, and a sedentary lifestyle, all of which contribute to physiological dysregulation, inflammation, and an increased risk of metabolic disorders (5, 6).

The results also highlighted regional differences in health outcomes, with individuals from certain European countries (i.e., Germany, and France) exhibiting lower odds of being at risk or with disease, potentially reflecting better healthcare systems, socioeconomic stability, and lifestyle behaviors in these regions. Germany and France, for instance, have well-established universal healthcare systems with extensive preventive care programs, which may contribute to lower disease prevalence and better overall health outcomes (99). Furthermore, these countries emphasize public health policies promoting PA and healthy eating habits, which could explain the observed protective effects. Beyond sociodemographic and lifestyle indicators, inter-country differences may also reflect broader contextual determinants, including food system resilience, economic disparities, cultural norms surrounding traditional foods, and national-level nutrition policies (34, 71–73). Variations in urban–rural food access, affordability of MedDiet components, and social support structures may likewise contribute to the observed heterogeneity. These structural factors should be considered when interpreting cross-country comparisons.

In terms of living environment, a simple proportional analysis of health status indicated a higher proportion of healthy individuals in urban environments compared to rural areas (78% vs. 66%). This finding aligns with previous research suggesting that rural areas often experience higher rates of chronic diseases due to limited healthcare access, lower socioeconomic status, and reduced public health infrastructure (15). However, a more in-depth MLR analysis, adjusted for age as a continuous variable, revealed that urban and suburban living were associated with significantly higher odds of being at risk or having diseases, suggesting that rural environments may offer protective health benefits. These contradictory findings within the same dataset highlight the importance of conducting deeper statistical analyses in health research, rather than relying solely on proportional distributions. Crucially, controlling for confounding variables such as age is indispensable when interpreting health-related outcomes, as overlooking these factors may lead to misleading conclusions. The MLR findings, which suggest that rural living may be more favorable for health outcomes, could potentially be explained by greater opportunities for PA, lower exposure to urban stressors, and stronger adherence to traditional dietary habits (15, 74).

One possible explanation for the observed urban health disadvantage lies in the negative health impacts of urbanization, including higher levels of air pollution, increased consumption of ultra-processed foods, sedentary lifestyles and chronic stress (14, 75), all of which contribute to higher risks of obesity, type 2 diabetes, and cardiovascular diseases (14). Moreover, urbanization often leads to food system transformations, with greater availability of processed and energy-dense foods and a shift away from traditional, nutrient-rich diets, further exacerbating the risk of NCDs (76).

At the same time, it is important to recognize that rural environments are not without health challenges. Despite their potential protective effect, a substantial proportion of at-risk and diseased individuals reside in these areas, with unadjusted proportions indicating that nearly two-thirds of the rural population falls into these health-risk categories, irrespective of age. While rural environments may offer lower exposure to urban stressors, environmental determinants such as climate change, air pollution, and resource scarcity disproportionately affect rural populations, potentially offsetting their health advantages and contributing to a significant health burden. Indeed, rural communities are often more vulnerable to extreme weather events, food insecurity, and limited healthcare accessibility, all of which contribute to chronic disease risk and disparities in health outcomes (76). Additionally, longer commuting distances and insufficient healthcare infrastructure can restrict access to preventive care services, leading to delayed diagnoses and poorer management of chronic conditions (99). Given the dual impact of urbanization on health, public health policies must focus on creating healthier urban environments while addressing the vulnerabilities of rural populations. Urban planning should prioritize green spaces, active commuting infrastructure, and stricter regulations on ultra-processed food marketing, while rural healthcare services require greater investments to improve accessibility and preventive care programs. Addressing these challenges requires integrated health strategies that consider both socio-environmental determinants and regional disparities to effectively mitigate NCD risks and promote long-term well-being across diverse populations.

Regarding the gender factor, this study found that males exhibited significantly higher odds of being at risk or having diseases compared to females, a finding consistent with prior research on gender-related health disparities. While biological factors play a role in differential disease susceptibility between men and women, behavioral and socio-cultural factors also contribute to these disparities. Men are generally less likely to seek preventive healthcare services, engage in routine medical check-ups, and adopt proactive health behaviors compared to women (5). Additionally, higher engagement in risk behaviors such as smoking and alcohol consumption among men contributes to their increased vulnerability to cardiovascular diseases, obesity, and metabolic disorders (77, 78). However, gender disparities in health are not uniform across populations and may be influenced by socioeconomic status, cultural norms, and healthcare accessibility. Research has shown that women from lower-income backgrounds, particularly in urban slums are at greater risk of metabolic disorders due to factors such as poor diet, lack of exercise, and limited access to healthcare services (100). Additionally, these women are more susceptible to sexually transmitted infections and unplanned pregnancies, further exacerbating health disparities (101). These findings emphasize the need for gender-specific health interventions that encourage preventive care uptake among men while addressing healthcare accessibility issues for women in socioeconomically disadvantaged groups.

The present study supports the well-documented protective effect of marriage on health outcomes, as married individuals exhibited better health status compared to those who were widowed, divorced, or separated. These findings align with research indicating that social support and shared health behaviors within a partnership contribute to lower stress levels, better mental well-being, and higher adherence to preventive healthcare measures (9, 10). Marriage has been associated with better dietary habits, greater PA levels, and reduced engagement in high-risk behaviors such as smoking and excessive alcohol consumption, factors that collectively contribute to improved health outcomes (11). Conversely, widowed and divorced individuals face greater health risks, likely due to higher stress, lower social support, and reduced adherence to healthy behaviors (10, 11). The loss of a partner often leads to increased loneliness, psychological distress, and reduced motivation for health-promoting activities, all of which contribute to elevated risks of NCDs, including cardiovascular disease, metabolic disorders, and depression (6). Moreover, widowhood in older adults is frequently associated with poor nutritional status and decreased PA, further exacerbating the risk of chronic disease progression (79).

Interestingly, while marital status was a significant predictor of health status in the model focusing on geo-demographic and socioeconomic factors, its effect became non-significant when the model incorporated psychological and lifestyle factors. This suggests that the protective effect of marriage may be partly mediated by psychological well-being and health-related behaviors. In other words, the benefits of marriage on health outcomes may stem from reduced psychological distress, greater social engagement, and healthier lifestyle choices, rather than marital status alone. When these additional factors are accounted for, marriage may no longer exert an independent effect on health, highlighting the complex interplay between social relationships, mental health, and behavioral factors in determining overall mental health.

Overall, these geo-demographic and socioeconomic patterns should be interpreted alongside the psychological and lifestyle domains identified in the final refined regression model (Model 8), which retained only variables that were significant moderators in the preceding models. The inclusion of BMI, anxiety, life satisfaction, insomnia, alcohol consumption, sitting time, and MedDiet adherence, alongside education, employment, gender, and living environment in this model, highlights the interdependent nature of behavioral, psychological, and socio-environmental factors in determining health status. Such integration supports the view that regional and cultural contexts may modulate individual lifestyle effects, underscoring the need for multi-level interventions that jointly address structural and behavioral determinants of health.

### 4.2 Mental health and sleep-related determinants

The Quade’s Rank ANCOVA results indicates that participants with poor health (either at risk or with diseases) exhibited significantly higher levels of perceived depression, anxiety, and stress, along with lower life satisfaction scores, suggesting a strong link between health deterioration and psychological distress. Sleep patterns were similarly associated with health status, with those in poor health experiencing worse sleep quality and higher insomnia severity, further exacerbating their overall well-being. Notably, across various regression models, anxiety, life satisfaction, sleep quality, and insomnia significantly contributed to MedLife adherence, with anxiety emerging as the strongest predictor, followed by insomnia, life satisfaction, and sleep quality.

The present findings underscore the critical role of psychological well-being in determining health status, with higher life satisfaction positively associated with better health outcomes, while stress, anxiety, and insomnia severity were linked to poorer health. These results align with a growing body of evidence demonstrating the bidirectional relationship between mental health and chronic disease risk (80). Psychological distress, particularly anxiety and chronic stress, has been implicated in systemic inflammation, metabolic dysfunction, and dysregulation of the hypothalamic–pituitary–adrenal (HPA) axis, all of which contribute to heightened risks of cardiovascular diseases, diabetes, and metabolic syndrome (6, 41). Furthermore, mental health disorders frequently coexist with chronic diseases, reinforcing a vicious cycle in which emotional distress exacerbates physiological deterioration, while chronic illness further fuels mental health decline (81, 98). The present study also highlights the prominent role of anxiety as the strongest predictor of health status, followed by insomnia, life satisfaction, and sleep quality. These findings reinforce the substantial impact of psychological distress on overall well-being, with higher anxiety levels significantly associated with poorer health outcomes. While mild anxiety may sometimes act as a motivator for health-related behaviors, a phenomenon often explained through the concept of implementation intentions (102), chronic anxiety and stress have been associated with detrimental health effects, including immune dysfunction, increased inflammation, and heightened vulnerability to NCDs (42). Beyond direct physiological effects, psychological distress also influences lifestyle adherence, compounding long-term health risks. Additionally, social and emotional well-being are deeply intertwined with health status, as evidenced by research linking loneliness and social isolation to adverse physical and mental health outcomes, including increased risks of cardiovascular disease, cognitive decline, and premature mortality (9, 40, 43). These associations are largely driven by heightened psychological stress, greater vulnerability to depression and anxiety, and the adoption of unhealthy coping behaviors such as physical inactivity, poor diet, and substance use (9). The present findings highlight the critical role of mental health interventions in disease prevention, emphasizing the need for integrated public health strategies that address psychological distress and its cascading effects on overall health. Prioritizing mental health support, stress management, and social engagement can help mitigate long-term health risks and enhance well-being.

In addition to psychological distress, sleep quality emerged as a significant factor in predicting health status, with poor sleep quality and higher insomnia severity associated with adverse health outcomes. These findings support prior research linking poor sleep patterns to metabolic disturbances, obesity, and cognitive decline (82, 83). Sleep is a fundamental regulator of physiological and cognitive function, and chronic sleep deprivation has been shown to disrupt glucose metabolism, increase inflammatory markers, and impair immune function, all of which contribute to heightened risks of hypertension, diabetes, and cardiovascular diseases (84). The modern lifestyle, characterized by excessive screen time, long working hours, and high-stress environments, has further contributed to widespread sleep disturbances, leading to increased prevalence of mental health disorders and cardiometabolic diseases (85). Moreover, the relationship between sleep and mental health is bidirectional, with poor sleep quality exacerbating psychological distress, while high levels of stress and anxiety contribute to sleep disturbances (84). Insomnia, in particular, has been linked to heightened emotional reactivity, impaired cognitive processing, and increased susceptibility to depression and anxiety (85). Given the significant influence of sleep-related factors on health status, our findings highlight the importance of incorporating sleep hygiene promotion into public health strategies. This includes limiting screen exposure before bedtime, establishing structured sleep routines, and addressing work-life balance challenges.

### 4.3 The role of BMI and lifestyle behaviors in health outcomes

This study confirms the importance of BMI and lifestyle factors, including smoking, alcohol consumption, dietary patterns, PA, and social participation, in shaping health status. The MLR analysis revealed significant associations between BMI and smoking with health status, showing that both obesity and underweight, as well as smoking behaviors, were linked to significantly higher odds of being at risk or having diseases. Findings from Quade’s Rank ANCOVA further demonstrated the association between health status and key lifestyle behaviors, with healthier individuals reported to be more socially engaged, spent less time sitting, and showed better dietary adherence. Additionally, healthy participants had significantly lower BMI, whereas individuals at risk and those with diseases exhibited progressively higher BMI levels.

Notably, across various regression models, BMI, sitting time, alcohol consumption, and adherence to the MediLife and its associated barriers (captured by the MBQ) were significant contributors to health status, with BMI emerging as the strongest predictor. Social participation was only significant in individual category-specific models, losing its significance in the comprehensive model. However, IPAQ scores showed limited associations across models, suggesting that the impact of PA behaviors may be more strongly mediated by sitting time rather than total activity levels.

The present findings highlight the strong association between dietary habits and health status, reinforcing prior evidence that a healthy diet, such as the MedDiet, plays a crucial role in disease prevention and overall well-being (31, 66, 74). The MedDiet, characterized by a high intake of fruits, vegetables, whole grains, and olive oil, has been extensively linked to reduced risks of cardiovascular disease, metabolic disorders, and neurodegenerative conditions (74, 86). These protective effects are largely attributed to its anti-inflammatory and antioxidant properties, which contribute to improved gut microbiome health, enhanced insulin sensitivity, and reduced oxidative stress (87, 88).

Despite the well-documented health benefits of the MedDiet, barriers to adherence remain significant, particularly among low-income populations where time constraints, affordability, and food accessibility heavily influence dietary choices (39). The increasing reliance on ultra-processed foods, driven by economic limitations, aggressive marketing, and evolving lifestyles, has contributed to rising obesity rates, cardiovascular disease risk, and premature mortality (14, 36). These findings align with recent data from Giampieri et al. (37), which demonstrated an inverse relationship between UPF consumption and MedDiet adherence in Mediterranean youth, emphasizing that adherence remains under threat even in traditional settings.

Recent findings (34, 35) further highlight key barriers to MedDiet adherence, including low motivation, time-consuming meal preparation, and unfavorable taste perceptions. Additionally, individual dietary preferences (e.g., veganism) and the labor-intensive nature of traditional Mediterranean recipes pose further challenges. Economic constraints also play a major role, with high food costs and limited availability negatively impacting adherence, particularly in urban–rural and low-income communities. To overcome these barriers, policy-driven interventions are essential, including subsidies for healthier foods, improved food distribution systems, and stricter regulations on ultra-processed food marketing. Such measures are crucial for improving dietary accessibility and thereby help reducing health disparities. Given that UPF consumption appears endemic in Mediterranean adolescents and often co-occurs with sedentary behavior and other lifestyle factors (30), interventions should target not only dietary education but holistic lifestyle shifts, including physical activity, screen-time limitation, and family-based meal practices.

Indeed, the balance between energy intake and expenditure is a key determinant of body weight and overall health. While dietary habits regulate energy intake, PA and sedentary behavior determine energy expenditure, together shaping metabolic health and disease risk. Although PA is a well-established protective factor against NCDs (16), our findings indicate that sitting time had a stronger association with health status than total PA levels. This suggests that sedentary behavior may exert a more dominant influence on health risks, a finding that aligns with prior research emphasizing the adverse effects of prolonged sitting on metabolic health (89, 96). A landmark study by Ekelund et al. (90) demonstrated that prolonged sitting time can negate the benefits of meeting the minimum PA recommendations (8.3–16.7 MET-hours per week). Specifically, individuals who sit for more than 8 h per day require more than 35.5 MET-hours per week to offset the increased mortality risks associated with prolonged sitting. This highlights the limitations of traditional PA guidelines, which may fail to account for individual sitting behaviors. Our results reinforce the need for more personalized PA recommendations, advocating for both increased movement and targeted strategies to reduce sedentary time. Encouraging breaks from prolonged sitting, workplace interventions, and urban planning initiatives that promote active commuting may be critical steps in mitigating the negative health effects of sedentary lifestyles.

As BMI is largely driven by the balance between energy intake (diet) and energy expenditure (PA), it is unsurprising that it emerged as the strongest predictor of health status. This finding is consistent with the well-documented association between elevated BMI and increased risk of various diseases. A comprehensive meta-analysis involving 239 prospective studies with 10.6 million participants across four continents found that both overweight and obesity are linked to higher all-cause mortality. Specifically, each 5 kg/m^2^ increase in BMI was associated with a 31% higher risk of all-cause mortality (91). Obesity is a key driver of systemic inflammation, insulin resistance, and cardiovascular dysfunction, predisposing individuals to diabetes, hypertension, and other metabolic disorders (92), reflecting the critical role of metabolic regulation in overall well-being (14). Similarly, underweight status has been associated with nutritional deficiencies, weakened immune function, and increased frailty, particularly in older adults (103, 104). The progressive increase in BMI levels among at-risk and diseased individuals further reinforces the importance of maintaining a healthy weight through balanced nutrition and active lifestyles.

Public health strategies should focus on promoting sustainable weight management approaches rather than restrictive dieting, ensuring that interventions address both dietary quality and PA levels while reducing sedentary behaviors. By targeting modifiable lifestyle factors, effective policies can help mitigate the long-term metabolic and psychological consequences of unhealthy weight trajectories.

The present findings also support the detrimental impact of smoking and alcohol consumption on health status, particularly when these behaviors co-occur with other unhealthy lifestyle factors. Smoking is well established as a major contributor to oxidative stress, impairing vascular function, and promoting systemic inflammation (105), all of which contribute to a heightened risk of cardiovascular diseases, cancer, and respiratory conditions. Additionally, smoking often interacts with other risk factors, such as poor diet, physical inactivity and psychosocial stress (106), amplifying overall disease susceptibility. Similarly, excessive alcohol consumption has been linked to liver disease, metabolic dysfunction, disturbances, and increased risk of cardiovascular events, with its negative effects further exacerbated in individuals with coexisting unhealthy behaviors (93, 107). The negative associations observed in our regression models further support prior research demonstrating that even moderate alcohol intake may contribute to increased disease risks over time (108). These findings highlight the urgent need for continued public health campaigns aimed at reducing smoking and alcohol consumption, particularly among high-risk populations.

Beyond physiological and behavioral risk factors, social participation emerged as an important determinant of health status, reinforcing previous findings that social engagement reduces risks of depression, cardiovascular disease, and all-cause mortality (40). Social participation encourages positive health behaviors, fosters emotional resilience, and enhances mental well-being, ultimately contributing to better long-term health outcomes. However, in the comprehensive regression model, social participation lost its significance, suggesting that its effects on health status may be mediated through other factors, such as psychological well-being and lifestyle choices. The modern trend toward increased social isolation, exacerbated by digital lifestyles and post-pandemic behavioral shifts, raises concerns about its long-term implications on public health (94, 109). Addressing these concerns requires community-based interventions, policies that promote social inclusion, and urban designs that encourage interpersonal engagement.

Taken together, these findings reinforce the importance of balanced nutrition, active lifestyles, and social engagement in maintaining good health, while also highlighting the exaggerated health risks associated with obesity, smoking, and alcohol consumption; emphasizing the need for multi-level public health interventions (97).

### 4.4 Strengths, limitations, and future perspectives

This study benefits from its multinational design, standardized methodology, and large sample size, enhancing cross-regional comparability and statistical power. A key strength is the structured regression approach, which systematically identified the most robust predictors of health status, while controlling for potential confounders like age ensured unbiased results. However, several limitations should be considered. First, its cross-sectional design prevents causal interpretations. Second, health status was self-reported by participants based on well-defined criteria, which enhances consistency but lacks biological confirmation. Third, reliance on self-reported data for lifestyle, dietary, and psychological factors may be subject to recall and social desirability biases. Fourth, selection bias may have occurred, as participants were recruited via online channels and may over represent individuals with internet access and higher education. Although the models included major socioeconomic status indicators (e.g., education, employment status, and living environment) together with age, gender, and region, harmonized income and detailed occupational classifications were not available across countries. This limitation is common in cross-national surveys, where education and employment are considered the most reliable and comparable socioeconomic measures. Consequently, residual confounding by unmeasured detailed socioeconomic dimensions cannot be fully excluded. Additionally, while key associations were identified, the underlying mechanisms remain speculative, relying on existing literature rather than direct physiological measurements. Future research should employ longitudinal and experimental designs with objective measurements to confirm causality and elucidate the mechanisms linking lifestyle, psychological, and health outcomes. Notably, our findings align with conceptual frameworks such as that proposed by Hemmingsson (95), in which financial strain can contribute to family stress and psychological distress, which in turn may foster maladaptive coping behaviors (e.g., sedentariness, unhealthy diet, poor sleep). These mechanisms have been proposed to underlie socioeconomic disparities in obesity and related health outcomes. Building on this model, future studies should aim to quantify these pathways and test potential mediating and moderating effects to better understand how socioeconomic stress and mental health interact with lifestyle behaviors in shaping overall health status. Tailored, context-sensitive strategies are also needed to promote active and healthy lifestyles across diverse populations.

## 5 Conclusion

This large multinational study highlights the complex and multifactorial interplay between psychological, socioeconomic, lifestyle, and environmental factors in determining health status. The findings demonstrate that anthropometric factors, psychological well-being, sleep characteristics, and lifestyle behaviors collectively contribute to health outcomes. BMI emerged as the strongest predictor of health status, followed by psychological distress, particularly anxiety, insomnia severity, alcohol consumption, and sedentary behavior. Conversely, higher life satisfaction, education level, sleep quality, and adherence to the Mediterranean lifestyle were consistently associated with better health status. In addition to these individual determinants, geo-demographic and socioeconomic conditions such as education and employment further shaped health outcomes.

These findings reinforce that effective prevention strategies should address multiple determinants simultaneously rather than focusing on single risk factors. The results support the need for multi-level public health strategies integrating mental health support, sleep education, dietary and physical activity interventions, and reduction of sedentary behavior, while promoting adherence to the Mediterranean lifestyle. Social participation and awareness initiatives may further support the adoption of sustainable health-promoting behaviors.

Given the growing burden of non-communicable diseases and lifestyle-related health risks, policymakers should prioritize community-based interventions, targeted awareness campaigns, and policy-driven strategies to reduce health disparities and promote equitable access to healthy lifestyles. Considering the observed cross-country variability, interventions should be adapted to local socioeconomic and cultural contexts. To support evidence-based policymaking, a detailed Public Health Implications section, including recommendations for targeted interventions derived from the present findings, is provided as Supplementary material.

Future research should explore causal mechanisms underlying the observed associations through longitudinal and intervention-based designs. Such approaches will allow a deeper understanding of health determinants and support the development and evaluation of targeted preventive strategies.

## Data Availability

The raw data supporting the conclusions of this article will be made available by the authors, without undue reservation.

## Footnotes

1 accessible at: www.soscisurvey.de/en/privacy
