# Supplementary material for: Multifactorial determinants of health status: insights from the MEDIET4ALL large-scale survey on eco-sociodemographic, psychological, and lifestyle (diet, physical activity, and sleep) factors
Source: Front Public Health. 2026 Apr 10;14:1704240. doi: 10.3389/fpubh.2026.1704240 (PMC13106316; doi:10.3389/fpubh.2026.1704240)
Supplement: Supplementary file 1 [file Supplementary_file_1.docx]

**Supplementary material: Public Health Implications**

The findings of this study demonstrate that health status is influenced by a combination of anthropometric, psychological, lifestyle, and socioeconomic factors. The strongest predictors of poorer health status included higher BMI, anxiety, insomnia severity, alcohol consumption, and prolonged sitting time, while higher life satisfaction, education level, sleep quality, and adherence to the Mediterranean lifestyle were associated with better health outcomes. These results provide an empirical basis for targeted public health strategies focusing on modifiable determinants of health across diverse populations..

To address these complex and interconnected determinants, multi-level public health interventions must adopt a holistic, preventive, and policy-driven approach. The following areas for intervention are particularly critical:

***Health Literacy and Education Initiatives***

Higher education level was consistently associated with better health status in the regression models, indicating that health literacy and awareness may play an important protective role.. Policymakers should prioritize:

- Integrating health education into school curricula to promote lifelong healthy habits.
- Developing nationwide health literacy programs, particularly in low-income and rural areas, to improve awareness of nutrition, PA, sleep, and preventive healthcare.
- Community and digital health education initiatives may support the adoption of healthier lifestyle behaviours identified as protective factors in this study.

***Reducing Socioeconomic Barriers to Healthcare and Healthy Lifestyles***

Employment status was significantly associated with health status, with unemployed individuals showing higher odds of being at risk or having diseases. These findings suggest that socioeconomic conditions may influence health through their effects on lifestyle behaviours and psychological well-being. Potential strategies include:

- Improving access to preventive healthcare services for socioeconomically vulnerable groups.
- Supporting initiatives that facilitate adoption of healthy lifestyles among unemployed and economically disadvantaged populations.
- Developing community-based programs that address combined socioeconomic and lifestyle risk factors.

***Addressing the Dual Burden of Urban and Rural Health Challenges***

This study revealed contradictory health trends in urban and rural environments, highlighting both advantages and challenges in each setting. Targeted, region-specific health strategies are required:

- In urban environments, strategies that reduce sedentary behavior and promote active lifestyles may be particularly relevant.
- In rural environments, improving access to preventive health services may help reduce existing health risks.

***Promoting Healthy Lifestyles Through Policy-Driven Interventions***

*Enhancing Dietary Accessibility and Affordability*

Adherence to the Mediterranean lifestyle was positively associated with health status, while dietary barriers and alcohol consumption were negatively associated with health outcomes. Thereby, food system transformations are essential to promote healthier eating patterns:

- Subsidizing fresh, minimally processed foods to improve accessibility and affordability, particularly for low-income and rural populations.
- Imposing stricter regulations on ultra-processed food marketing, especially targeting children and adolescents.
- Encouraging food reformulation strategies to reduce added sugars, trans fats, and sodium in packaged foods.
- Promoting food labeling transparency to help consumers make informed dietary choices.

*Reducing Sedentary Behavior and Personalizing Physical Activity Recommendations*

Sitting time was a significant negative predictor of health status and showed stronger associations with health outcomes than physical activity levels. These findings highlight the importance of reducing sedentary behaviour in addition to promoting physical activity. Policymakers should:

- Develop new PA recommendations that integrate sitting time reductions alongside moderate-to-vigorous PA.
- Encourage "movement-friendly" workplaces by implementing standing desks, active breaks, and walking meetings.
- Support public awareness campaigns addressing sedentary behaviour may complement physical activity promotion.
- Support PA programs tailored for high-risk individuals, emphasizing age, health condition, and occupation-specific exercise strategies.

*Implementing Comprehensive Smoking and Alcohol Reduction Strategies*

Tobacco use and excessive alcohol consumption were significantly associated with poorer health outcomes, highlighting the urgent need for aggressive reduction policies:

- Increasing taxation and price controls on tobacco and alcohol products to discourage consumption.
- Expanding smoking cessation programs and integrating behavioral interventions for alcohol reduction into routine healthcare.
- Regulating alcohol marketing and availability, particularly in high-risk populations.
- Educating the public on the cumulative effects of smoking and alcohol when combined with poor diet and sedentary behaviors.

***Addressing Psychological and Sleep-Related Health Risks***

Psychological factors, particularly anxiety and life satisfaction, together with sleep characteristics such as insomnia severity and sleep quality, were among the strongest predictors of poor health status, reinforcing the critical need for public health strategies targeting psychological well-being. Key interventions include:

- Expanding mental health services, particularly in primary care settings, to ensure early detection and management of anxiety, depression, and stress-related conditions.
- Implementing workplace mental health programs, offering stress management training and psychological support.
- Promoting community-based initiatives to reduce loneliness and social isolation, which are significant contributors to psychological distress.
- Enhancing public awareness about the importance of sleep hygiene, encouraging structured sleep routines, reduced screen exposure before bedtime, and improved work-life balance to combat sleep deprivation.

***Strengthening Social and Community Engagement***

Although social participation showed positive associations with health in some analyses (e.g., regression model 5), its contribution was not retained in the final comprehensive regression model. Nevertheless, community engagement initiatives, including group-based physical activity programs and neighborhood-based activities (particularly for older adults and high-risk groups) may strengthen social support networks and contribute to the effectiveness of broader lifestyle interventions

***Integrating Multi-Level Public Health Policies for Long-Term Impact***

Given the interconnected nature of health determinants identified in this study, comprehensive/multidisciplinary public health strategies addressing lifestyle, psychological, and socioeconomic factors simultaneously may provide the greatest benefit. Policymakers may:

- Adopt cross-sectoral collaborations between health, education, urban planning, and economic sectors to create integrated health-promoting environments.
- Utilize data-driven monitoring approaches may help evaluate intervention effectiveness across different populations.
- Encourage participatory decision-making, involving community stakeholders, healthcare professionals, and researchers in shaping policies tailored to diverse population needs.
